# Treating a Dentoalveolar Fracture in a Four-Year-Old Girl With a Functional Removable Partial Denture

**DOI:** 10.7759/cureus.65224

**Published:** 2024-07-23

**Authors:** Ishani Rahate, Punit Fulzele, Dhruvi Solanki, Nilima R Thosar, Sudhanwa N Deshmukh

**Affiliations:** 1 Department of Pediatric and Preventive Dentistry, Sharad Pawar Dental College and Hospital, Datta Meghe Institute of Higher Education and Research (Deemed to be University), Wardha, IND; 2 Pediatric Dentistry, Sharjah Specialized Dental Center, Sharjah, ARE

**Keywords:** avulsion, tooth injury, primary teeth, trauma, mandibular cortical fracture

## Abstract

In children and young adults, traumatic dental injuries are common. Children's tooth loss is mostly caused by dentoalveolar trauma. Owing to anatomical variations and developmental phases, treating such injuries is difficult. Trauma to deciduous teeth might harm the permanent tooth beneath; however, trauma to permanent teeth can worsen their long-term outlook. An alveolar segment trauma may result in the irreversible loss of dental tissues, malalignment, and deformity. The periodontium and pulpal tissues suffer significant harm as a result of this. In the current case, there was a segmental cortical fracture in the mandible of a four-year-old girl child.

## Introduction

Trauma associated with tooth socket joint is a major concern, particularly in younger children, since it can result in early loss of teeth that impairs normal oral function, appearance, and self-confidence, and changes the child's long-term treatment plan [1]. Smaller jaw sizes, centers of active bone development, and unerupted permanent teeth make it more difficult to make decisions for the management of fractures in children than in adults. The diagnosis of fracture of mandibular and maxillary alveolar parts should be considered based on a thorough clinical assessment and radiographic confirmation. Dentoalveolar fractures may result from facial trauma [1,2]. They can have a variety of causes depending on demography, sex, and age. Both young permanent teeth and primary teeth are most commonly fractured due to falls. Alveolar fractures can also be linked to the traumatized displacement of primary teeth [2,3].

Numerous challenges arise while managing fractures in primary and mixed dentition. First, some permanent teeth do not emerge until an individual is at least six years old [3]. In cases of mandibular dentoalveolar fractures, splinting is considered in children as they have smaller jaws, active growth centers present in the mandibular development that must remain intact, and close proximity of the primary teeth to the permanent tooth buds and inferior alveolar nerves [4,5].

It is difficult to save the anterior traumatized teeth through splinting which increases the chances of ankylosis. In the current case, mandibular cortical segmental fracture was associated with mobility of the anterior segment of teeth [6,7]. Dental trauma can happen at any time, and there should be awareness regarding such trauma in parents as well as school teachers [7-9].

## Case presentation

A four-year-old girl presented to the outpatient clinic of the pediatric dental department about 72 hours after trauma to her lower anterior teeth with the loss of a tooth in a sports accident. No significant medical history was provided. However, lacerations on the inferior border of the lip and mild displacement of the mandibular deciduous upper front incisors were observed. The child's health and family background were unremarkable. A tetanus vaccination was also administered to the patient. The child was healthy and fit after a general examination conducted at the department. The temporomandibular joint appeared to be functioning normally throughout the extraoral evaluation; there was no clicking or mandibular deviation.

**Figure 1:**
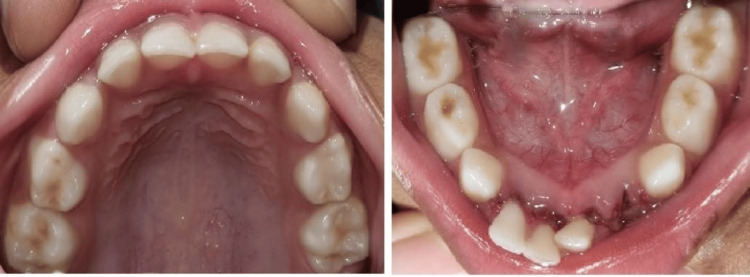
Preoperative intraoral photographs of maxillary and mandibular arch

**Figure 2:**
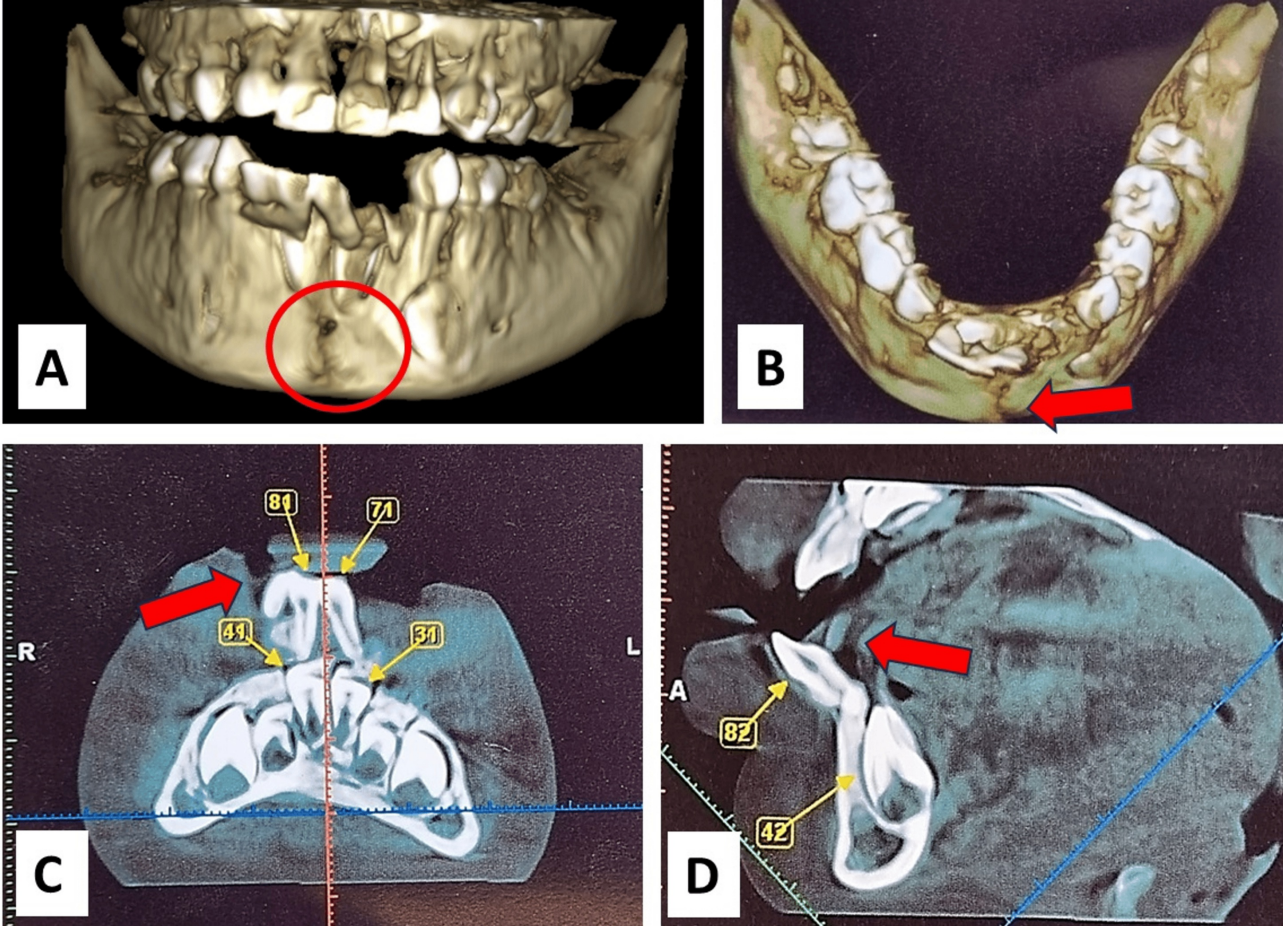
CBCT Images showing (A) Discontinuity of alveolus extending from 71, 81 region to lower border of mandible; (B) Mid symphysis fracture in lower jaw; (C) Missing tooth in 72; (D) Displacement of teeth in 71, 81 CBCT: cone-beam computed tomography

The child had a typical mouth opening. Gingiva's consistency and color were normal in the intraoral examination. Carious lesions were not discovered. In 71, 81, and 82, there was tenderness with vertical percussion; in 72, there was an avulsion, but the fracture line was evident clinically (Figure 1). For radiographic investigation, cone-beam computed tomography (CBCT) was done (Figure 2). Grade III mobility was associated with 71, 81, and 82, showing fracture at the mid-symphysis area of the lower jaw.

**Figure 3:**
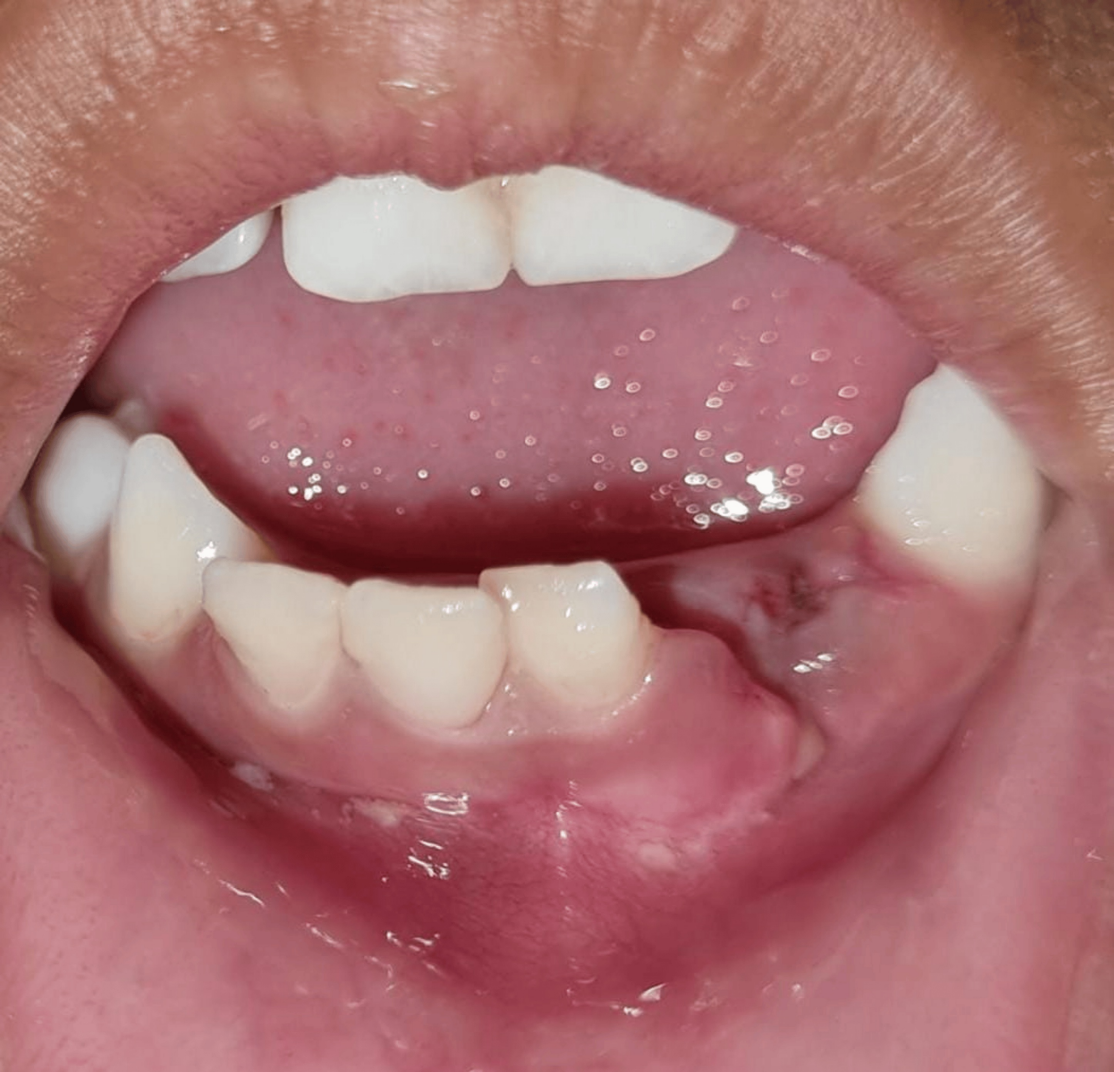
Fracture fragment associated with 71, 81, and 82

**Figure 4:**
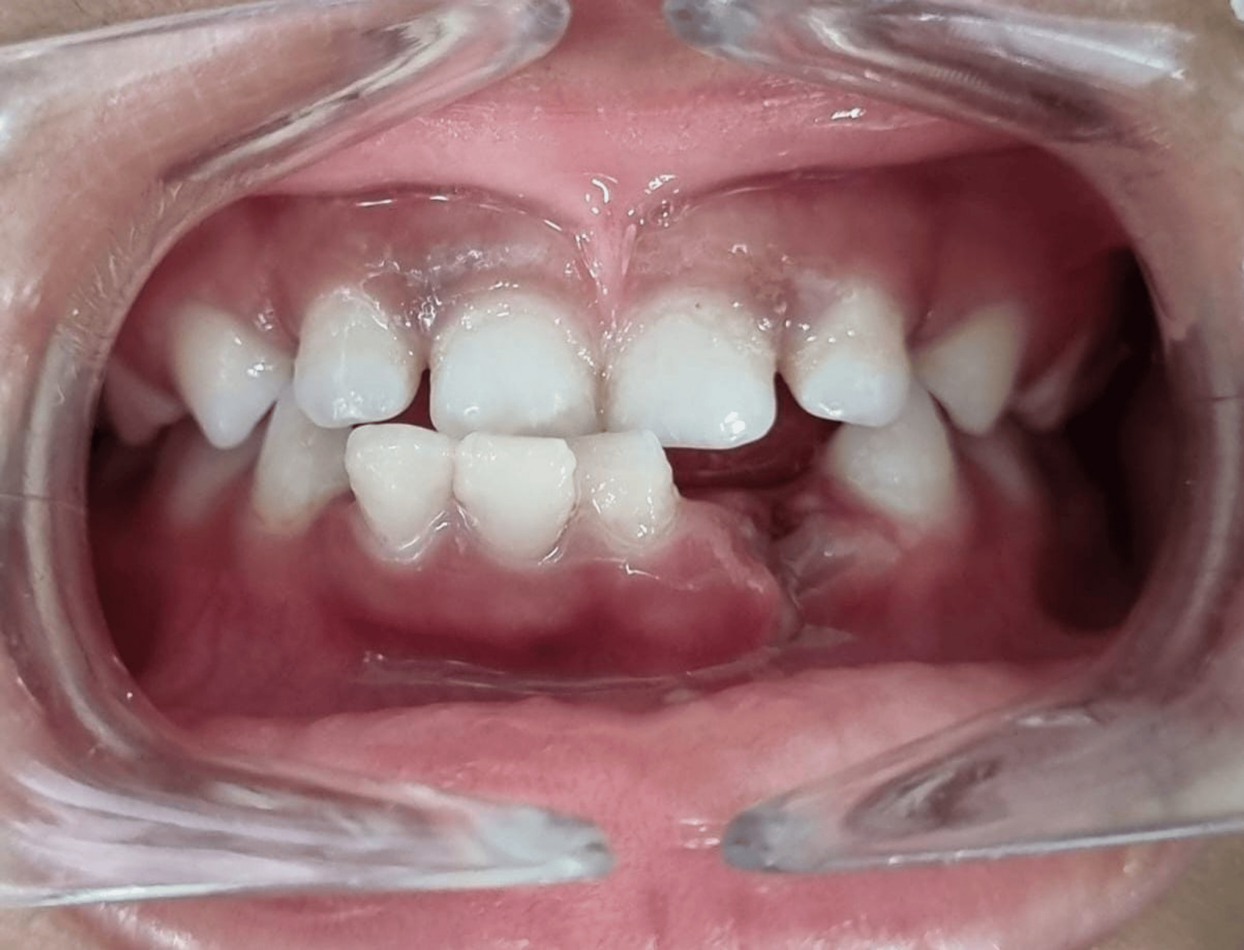
Dentoalveolar fracture at mid-symphysis region of mandibular arch

The lower cortical plate was movable. An occlusal examination revealed the mesial step primary molar relation. The clinical examination determined the dentoalveolar fracture with severe displacement of the cortical plate with subluxation of 71, 81, and 82 on the right side and avulsion of 72, mandibular gingival tissue damage, laceration of the inferior lip with alveolar fracture of the anterior region of the lower jaw (Figures 3, 4).

Consequently, a comprehensive assessment was done and a treatment plan was made. As the child was four years old and the lower anterior teeth were mobile, splinting of primary teeth was not preferred which could result in ankylosis. Hence, the extraction of mobile teeth was planned. After administering local anesthesia containing 2% lignocaine with 1:80,000, the mouth cavity was disinfected with a 0.12% chlorhexidine solution. Extraction was then performed for 71, 81, and 82, suture was placed with approximation at the site.

**Figure 5:**
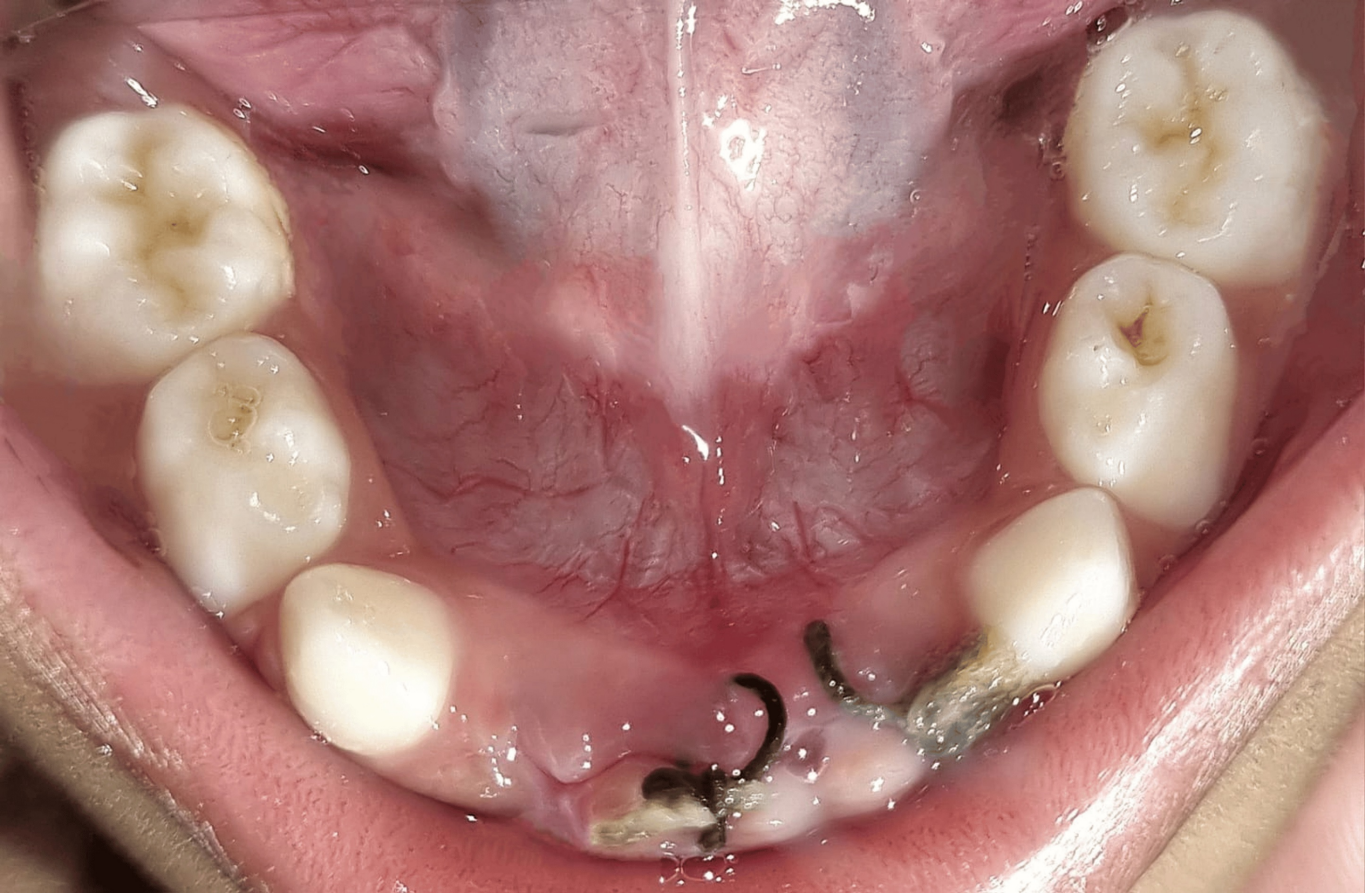
Postoperative intraoral photograph at the one-week follow-up

**Figure 6:**
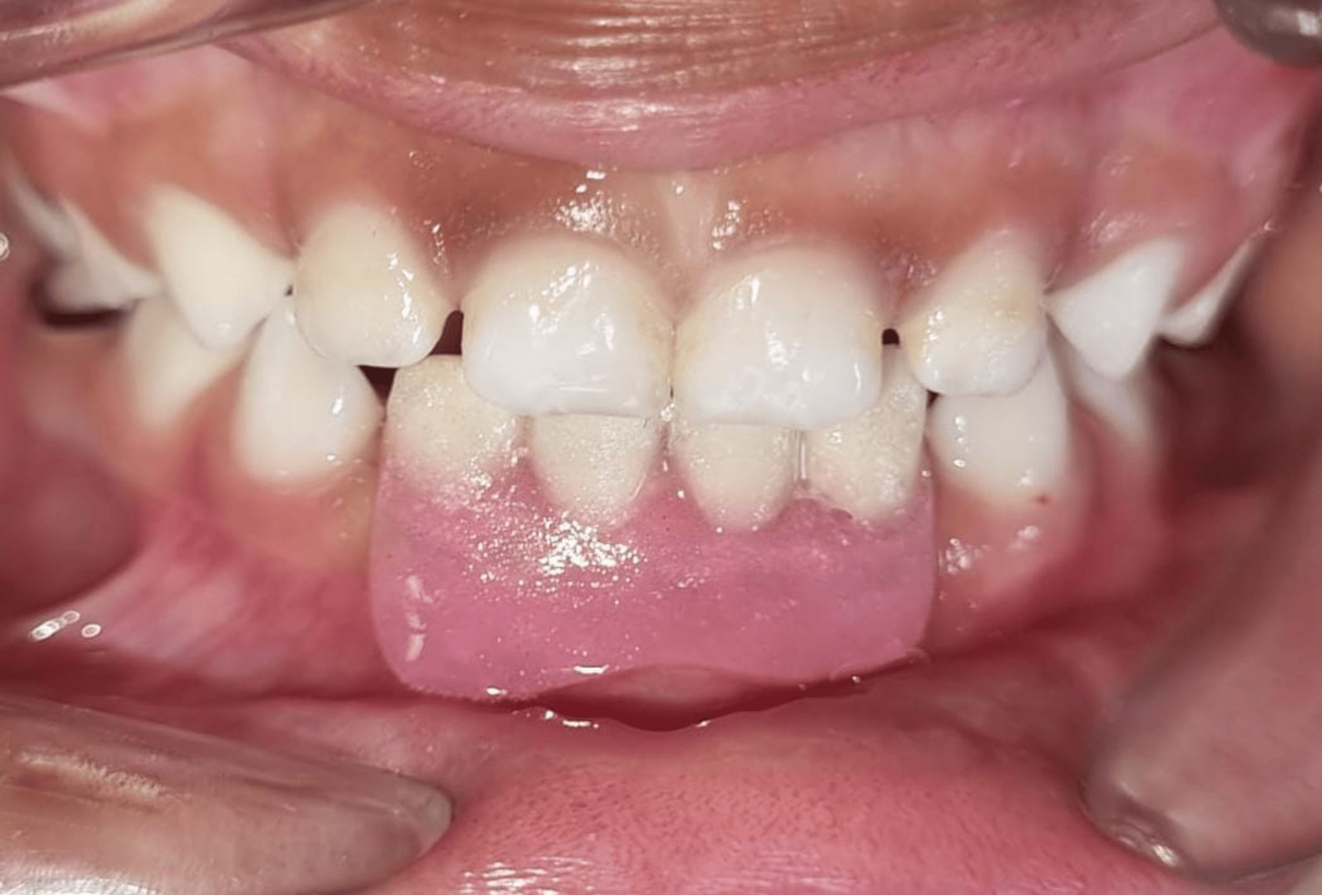
Postoperative labial view of removable partial space maintainer with lower arch

**Figure 7:**
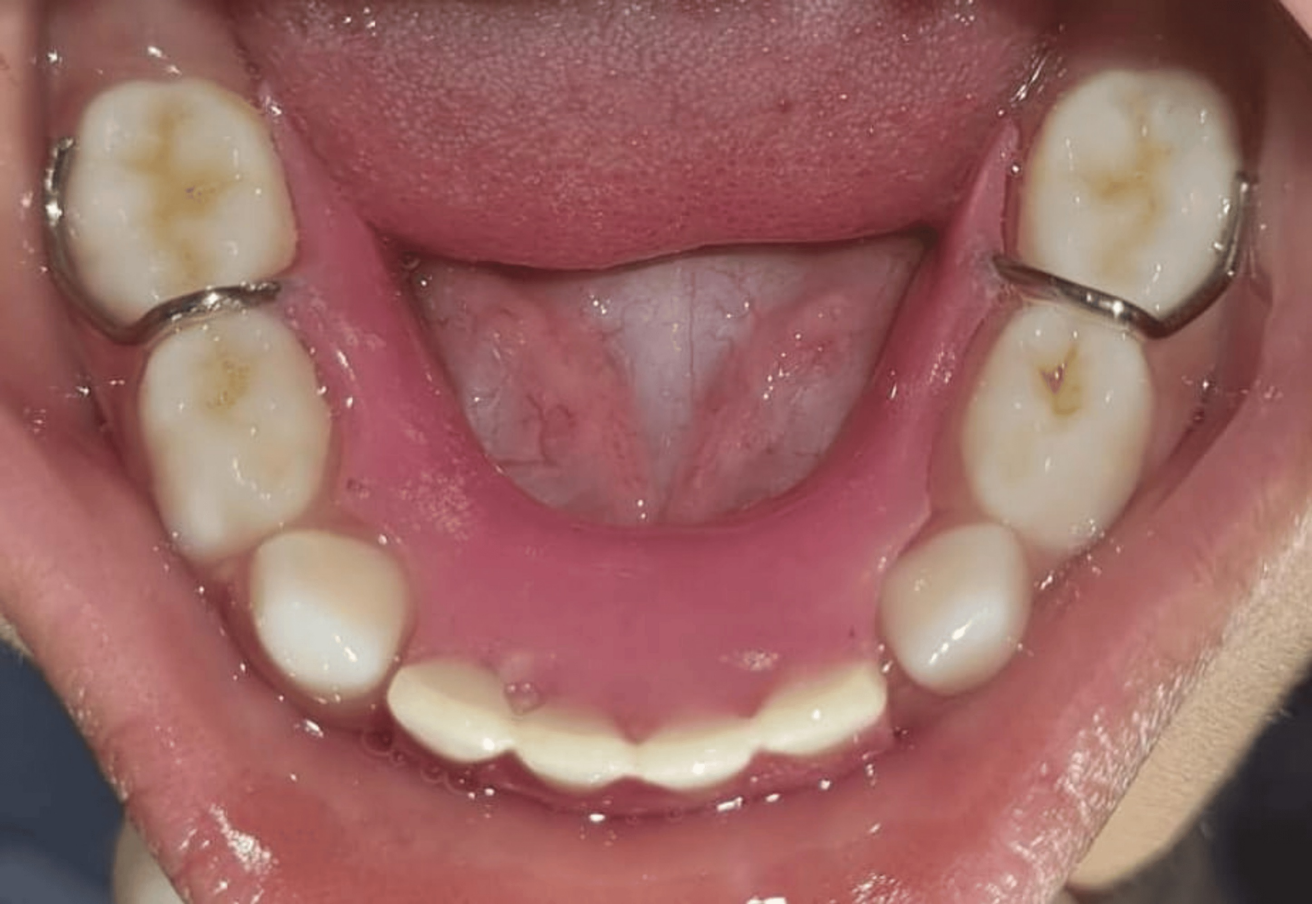
Postoperative mandibular arch view showing functional removable space maintainer

In this case, seven days following the injury, a follow-up clinical examination was performed to remove the sutures (Figure 5). Prosthetic rehabilitation was planned after complete healing. Tray selection was done for the upper and lower arch which was found to be U0. Using the fast set alginate impression material, the negative impression of the upper maxillary and lower mandibular arch was made and immediately the cast was poured with type III Gypsum product (dental stone) with the appropriate water:stone ratio. In the lower cast, the C clasp was fabricated using a 19 gauge wire with 75 and 85. The functional removable partial denture was made using cold-cure acrylic resin. Figures 6, 7 show postoperative images of the mandibular arch with a removable partial denture and space maintainer. The patient is recalled after every three months.

## Discussion

In this report, a sports accident resulted in a complicated trauma affecting dentoalveolar structures and soft tissues in a four-year-old child patient. A similar incident occurred with a 4.5-year-old girl child with a fall accident that resulted in a complicated trauma affecting dentoalveolar structures and soft tissues [9]. In another case by Gupta et al., the primary mandibular lower incisor of a 4.5 year-old-girl got entangled in the ring of the father’s hand [10].

The amount of time that has passed after an injury may influence the therapy plan. Readjusting teeth becomes more challenging after 48 hours of injury, according to Andreasen et al. [11]. For the current case, extraction was carried out around 72 hours after the trauma. However, despite the late presentation, treatment had satisfactory outcomes. According to several publications, this treatment method of repositioning or reimplantation of teeth is not as successful for primary teeth as it is for permanent incisor replantation [12]. There's a chance that it might harm the permanent tooth that replaces the lost one.

A primary incisor avulsion often results in lip lacerations, gingival tissue damage, luxation injuries to neighboring teeth, and facial bone fractures. Following an accident, there are three alternative courses of action: (i) Reimplantation of the extracted tooth, (ii) Prosthetic teeth to replace the lost teeth, or (iii) No treatment at all [12,13]. According to the International Association of Dental Traumatology guidelines, avulsed primary teeth should not be replanted in a young child as it proves to be a significant treatment burden including replantation, splint placement and removal, and root canal treatment and it can cause further damage to the permanent tooth or to its eruption [14]. Additionally, some researchers claim that it is absurd to replace deciduous teeth because doing so may cause infections in the jawbone that houses the permanent tooth or even in the actual tooth itself [15]. The deciduous tooth (replanted) may eventually become necrotic, uncomfortable, and may even develop an infection in its socket. It is rare for the central incisors of the mandible to avulsion. Relieving discomfort, avoiding potential harm to the growing permanent teeth, and reducing the potential risk are the key goals of diagnosing and treating traumatic dental injuries in children with primary dentition [13]. Preserving the permanent tooth below and determining the risk-benefit ratio are the main concerns when it comes to primary tooth avulsion. In routine clinical practice, the avulsion of deciduous teeth is less commonly seen than permanent teeth as the developmental growth of the successor tooth depends on its eruptive movement [5].

Avulsions, subluxations, and tooth fractures linked to fractures in the alveolar bone represent instances of trauma or fractures causing disruption in the integrity of tooth structure and alveolar bone [5]. Dentoalveolar fractures can cause many teeth to move or oscillate in a single segment, cut the gingiva and lip vermilion, injure the gingiva and surrounding hematoma, cause pain along the fracture line, and cause swelling or damage to the chin [16]. The fracture section should be replaced and the fracture fixated till the bone heals as part of the treatment for dentoalveolar fractures. One can use either closed reduction or open reduction to reduce fracture segments. Permanent wire, interdental wiring, arch bars, and acrylic splints are a few techniques for repairing broken bone segments [16,17]. Dentoalveolar fractures in children do not require splinting because of their quick bone healing process, which prevents the displacement of tooth segments or bone discomfort along the fracture line, chin injury, or edema [17].

## Conclusions

Successful treatment for dentoalveolar trauma patients depends on effective care. In this case, the ability to restore function, appearance, and parental satisfaction was achievable due to conservative care provided. This approach not only addresses the physical aspects of the injury but also restores the child's confidence.
